# Derivation and external validation of predictive models for invasive mechanical ventilation in intensive care unit patients with COVID-19

**DOI:** 10.1186/s13613-024-01357-4

**Published:** 2024-08-21

**Authors:** Gabriel Maia, Camila Marinelli Martins, Victoria Marques, Samantha Christovam, Isabela Prado, Bruno Moraes, Emanuele Rezoagli, Giuseppe Foti, Vanessa Zambelli, Maurizio Cereda, Lorenzo Berra, Patricia Rieken Macedo Rocco, Mônica Rodrigues Cruz, Cynthia dos Santos Samary, Fernando Silva Guimarães, Pedro Leme Silva

**Affiliations:** 1 Laboratory of Pulmonary Investigation, Institute of Biophysics Carlos Chagas Filho, Centro de Ciências da Saúde, Federal University of Rio de Janeiro, Avenida Carlos Chagas Filho, 273, Bloco G-014, Ilha do Fundão, Rio de Janeiro, 21941-902 RJ Brazil; 2 Pedro Ernesto University Hospital, State University of Rio de Janeiro, Rio de Janeiro, Brazil; 3 AAC&T Research Consulting LTDA, Curitiba, Brazil; 4 Department of Cardiorespiratory and Musculoskeletal Physiotherapy, Faculty of Physiotherapy, Federal University of Rio de Janeiro, Rio de Janeiro, Brazil; 5 Department of Anesthesia, Critical Care and Pain Medicine, Massachusetts General Hospital, Boston, MA USA; 6 Department of Anesthesia, Critical Care and Pain Medicine, Harvard Medical School, Boston, MA USA; 7 School of Medicine and Surgery, University of Milano-Bicocca, Monza, Italy; 8 Department of Emergency and Intensive Care, Fondazione IRCCS San Gerardo dei Tintori, Monza, Italy; 9 Respiratory Care Department, Massachusetts General Hospital, Harvard Medical School, Boston, MA USA; 10 Evandro Chagas National Institute of Infectious diseases, Oswaldo Cruz Foundation, Rio de Janeiro, Brazil

**Keywords:** COVID-19, SOFA score, SpO_2_, Multiple logistic regression, Invasive mechanical ventilation, External validation

## Abstract

**Background:**

This study aimed to develop prognostic models for predicting the need for invasive mechanical ventilation (IMV) in intensive care unit (ICU) patients with COVID-19 and compare their performance with the Respiratory rate-OXygenation (ROX) index.

**Methods:**

A retrospective cohort study was conducted using data collected between March 2020 and August 2021 at three hospitals in Rio de Janeiro, Brazil. ICU patients aged 18 years and older with a diagnosis of COVID-19 were screened. The exclusion criteria were patients who received IMV within the first 24 h of ICU admission, pregnancy, clinical decision for minimal end-of-life care and missing primary outcome data. Clinical and laboratory variables were collected. Multiple logistic regression analysis was performed to select predictor variables. Models were based on the lowest Akaike Information Criteria (AIC) and lowest AIC with significant *p* values. Assessment of predictive performance was done for discrimination and calibration. Areas under the curves (AUC)s were compared using DeLong’s algorithm. Models were validated externally using an international database.

**Results:**

Of 656 patients screened, 346 patients were included; 155 required IMV (44.8%), 191 did not (55.2%), and 207 patients were male (59.8%). According to the lowest AIC, arterial hypertension, diabetes mellitus, obesity, Sequential Organ Failure Assessment (SOFA) score, heart rate, respiratory rate, peripheral oxygen saturation (SpO_2_), temperature, respiratory effort signals, and leukocytes were identified as predictors of IMV at hospital admission. According to AIC with significant *p* values, SOFA score, SpO_2_, and respiratory effort signals were the best predictors of IMV; odds ratios (95% confidence interval): 1.46 (1.07–2.05), 0.81 (0.72–0.90), 9.13 (3.29–28.67), respectively. The ROX index at admission was lower in the IMV group than in the non-IMV group (7.3 [5.2–9.8] versus 9.6 [6.8–12.9], *p* < 0.001, respectively). In the external validation population, the area under the curve (AUC) of the ROX index was 0.683 (accuracy 63%), the AIC model showed an AUC of 0.703 (accuracy 69%), and the lowest AIC model with significant *p* values had an AUC of 0.725 (accuracy 79%).

**Conclusions:**

In the development population of ICU patients with COVID-19, SOFA score, SpO2, and respiratory effort signals predicted the need for IMV better than the ROX index. In the external validation population, although the AUCs did not differ significantly, the accuracy was higher when using SOFA score, SpO2, and respiratory effort signals compared to the ROX index. This suggests that these variables may be more useful in predicting the need for IMV in ICU patients with COVID-19.

**ClinicalTrials.gov identifier:**

NCT05663528.

**Supplementary Information:**

The online version contains supplementary material available at 10.1186/s13613-024-01357-4.

## Introduction

The COVID-19 pandemic led to a surge in critically ill patients, affecting the availability of resources [1]. Despite declining hospitalization rates across age groups, certain populations, such as older adults, infants, and individuals with underlying medical conditions or disabilities, continue to be hospitalized at higher rates. Among these patients, some may progress to severe conditions requiring invasive mechanical ventilation (IMV). Throughout the pandemic, numerous studies developed models to predict the need for IMV in patients with COVID-19. Some studies utilized non-pandemic databases [2] or developed models during the pandemic without validation [3–6]; others conducted internal validation across different hospital settings [7, 8]. However, external validation is crucial for estimating model accuracy in diverse patient populations encountered in real clinical practice, facilitating generalization of the results. Including readily available variables in predictive models enhances their practical usefulness [4, 6]. For example, respiratory rate and derived SpO_2_ indexes have been identified as effective predictive variables for IMV [4]; however, certain factors, such as computed tomography imaging of the lungs, although important for stratification of disease severity, may not be prioritized during model development [6].

The study hypothesizes that incorporating clinical and physiological variables into predictive models can improve the detection of the need for IMV in Intensive Care Unit (ICU) patients with COVID-19. Therefore, the aim of this study was to develop prognostic models for predicting the need for IMV in ICU patients with COVID-19 and compare their performance with the Respiratory rate-OXygenation (ROX) index. In addition, the models were externally validated using an international database, ensuring their applicability across diverse patient populations in real-world clinical practice.

## Methods

### Study design and patients

This observational, retrospective investigation adhered to the guidelines outlined in the Strengthening the Reporting of Observational Studies in Epidemiology (STROBE) statement [9]. It was conducted in three hospitals in Rio de Janeiro, Brazil, including University Hospital Clementino Fraga Filho, Pedro Ernesto University Hospital, and Evandro Chagas National Institute of Infectious Diseases. The Research Ethics Committee of Pedro Ernesto University Hospital approved the study protocol on 3 December 2021 (CAAE: 31062620.0.1001.5259). This study was registered on ClinicalTrials.gov (NCT05663528).

The development population was obtained retrospectively by including data from ICU patients aged 18 years and older with a diagnosis of COVID-19 (positive SARS-CoV-2 real-time polymerase chain reaction) between 1 March 2020 and 30 August 2021 were screened. The exclusion criteria were patients who received IMV within the first 24 h of ICU admission, pregnancy, clinical decision for minimal end-of-life care and missing primary outcome data. The external validation population was obtained from an Italian database of patients with COVID-19 admitted to hospital in a similar period as the development population.

### Data collection for the development population

ICU data were collected to characterize the sample. The database included data on age, days of symptoms, sex, presence or absence of comorbidities (systemic arterial hypertension, diabetes mellitus, obesity, chronic kidney disease, acquired immunodeficiency syndrome, chronic obstructive pulmonary disease), Sequential Organ Failure Assessment (SOFA) score, systolic and diastolic blood pressure, heart rate, respiratory rate, peripheral oxygen saturation (SpO_2_), ROX index, defined as the ratio of oxygen saturation as measured by pulse oximetry/FiO_2_ to the respiratory rate, presence or absence of febrile status, presence or absence of signs of breathing effort, hematocrit, leukocytes, lymphocytes, platelets, sodium and potassium levels, urea, creatinine, and C-reactive protein levels. Respiratory effort was assessed clinically based on the use of accessory muscles, paradoxical chest movement, intercostal retractions, nasal flaring, and neck retraction. The primary outcome variable was the need (yes or no) for IMV in ICU patients with COVID-19. Secondary outcome variables included time under IMV (in days), length of stay in the hospital and ICU, and in-hospital mortality rate. All data were collected from electronic medical records using the hospital information system.

ICU patients with clinical symptoms of acute respiratory failure due to COVID-19 underwent computed tomography imaging to quantify the area of lung damage. If the patient presented with hypoxemia (partial pressure of oxygen [PaO_2_] ≤ 60 mmHg or SpO_2_ ≤ 88%), supplementary oxygen was started immediately at between 1 and 15 L/min (nasal cannula [1–6 L/min], oxygen face mask [7–9 L/min], or reservoir mask [10–15 L/min]). If the patient had SpO_2_ < 88% with 10 L/min in a reservoir mask, high-flow nasal oxygen (HFNO) was indicated. If the work of breathing and dyspnea were detected in the absence of a need for emergency endotracheal intubation (characterized by a lowered level of consciousness; Glasgow Coma Scale score < 8, SpO_2_ < 88%, intense respiratory effort with the use of accessory muscles, pneumothorax not drained, and cardiac arrest), the patient was started on non-invasive ventilation (NIV) (in cases of predominance of respiratory distress) through an interface (oronasal or full face mask) or HFNO (in cases of predominance of hypoxemia with PaO_2_ < 60 mmHg).

NIV was first applied continuously through mechanical ventilators (SERVO-S, SERVO-E [Gettingge], Puritan Bennet 840 [Covidien]). Time under NIV was reduced progressively until weaning. Supplementary oxygen therapy was given after NIV if necessary to maintain SpO_2_ > 90% via a nasal catheter (1–5 L/min), a simple oxygen mask (6–10 L/min), or a reservoir mask (6–15 L/min). HFNO (Vapotherm [Vapotherm] or Optiflow [Fisher & Paykel] device according to availability) was used according to the inspired fraction flow level and was reduced progressively until weaning.

Failure of non-invasive respiratory support was defined as the need for endotracheal intubation with IMV according to the following criteria: clinical decision by the medical team; hypoxemia (PaO_2_ ≤ 60 mmHg) or acidosis (pH ≤ 7.35); low level of consciousness; worsening of the work of breathing; cardiopulmonary resuscitation event; intolerance to therapy or a face mask, or other [10].

### Data collection for the external validation population

Data for the Italian external validation population was obtained after a formal request. A case report form was sent electronically. Italian local coordinators could complete it with clinical and laboratory predictors previously highlighted by our development population. Electronic clinical healthcare records acquired and stored by Noemalife Galileo Core-1.5.6.4.5 [srvnewGalileo] and Draeger Innovian 2006, 2014 Draeger version vf7.0.1.

### Statistical analysis

The sample size was determined at the beginning of data collection. Assuming an alpha level of 5%, the final adjustment of the model (*r*^2^) of 20%, 15 predictive candidate variables, and 50% of patients with no IMV and 50% with IMV, the total number of patients was 350. The sample size was calculated using Riley’s proposed method [11] in a routine written in the R environment (R Core Team, 2021).

The database was verified to avoid significant missing data, defined as < 25% of the total amount. After variables with more than 75% of total data were detected, multiple logistic regression analysis following the backward stepwise method of selection of predictor variables was conducted to find the best model [12]. The dependent variable was the need or not for IMV. The selection of prediction variables was made using two different approaches: (1) lowest Akaike Information Criteria (AIC) and (2) lowest AIC with significant *p* values (most simple modeling). The odds ratios (ORs) with 95% confidence intervals (CIs) and relative *p* values were provided for both criteria.

Participant characteristics and predictor information were described for both the development and external validation populations (overall and stratified according to IMV status). For descriptive summary statistics, variables are reported as means (standard deviation), medians (interquartile range [IQR], 25–75%), or absolute and relative frequencies, as appropriate. The predictor variables were compared using unpaired Student’s t test for normally distributed data, Mann-Whitney U test for non-normally distributed data, or χ^2^ test for categorical data.

The predictive performance for both prognostic models (AIC and AIC with significant *p* values) was assessed and the ROX index obtained from the development population was evaluated to do the discrimination (i.e., the model’s ability to differentiate between individuals who were endotracheally intubated and adapted to IMV and those who did not require IMV) and calibration (the agreement between predicted and observed IMV risks) in both populations [13]. Discrimination was assessed in both models by quantifying the area under the receiver operating characteristic curve (AUC), i.e., the c-statistic [14]. Once the data were normalized, the Youden criteria [15] were used to choose the best threshold for different combinations. The AUC was computed to identify optimal cutoff points, considering the natural data distribution and the sensitivity and specificity of clinical variables in discerning patients who underwent endotracheal intubation and were adapted to IMV. The AUC and its corresponding 95% CI were then presented as a measure of the predictive performance of the clinical variables. The AUCs were compared using the DeLong’s algorithm [16], implemented with the by roc.test function from the “pROC” package [17] in the R environment. All analyses were performed in the R 4.0.4 environment (R Core Team, 2021) and considered significant when *p* < 0.05.

## Results

### Characteristics of the COVID-19 development population

**Fig. 1:**
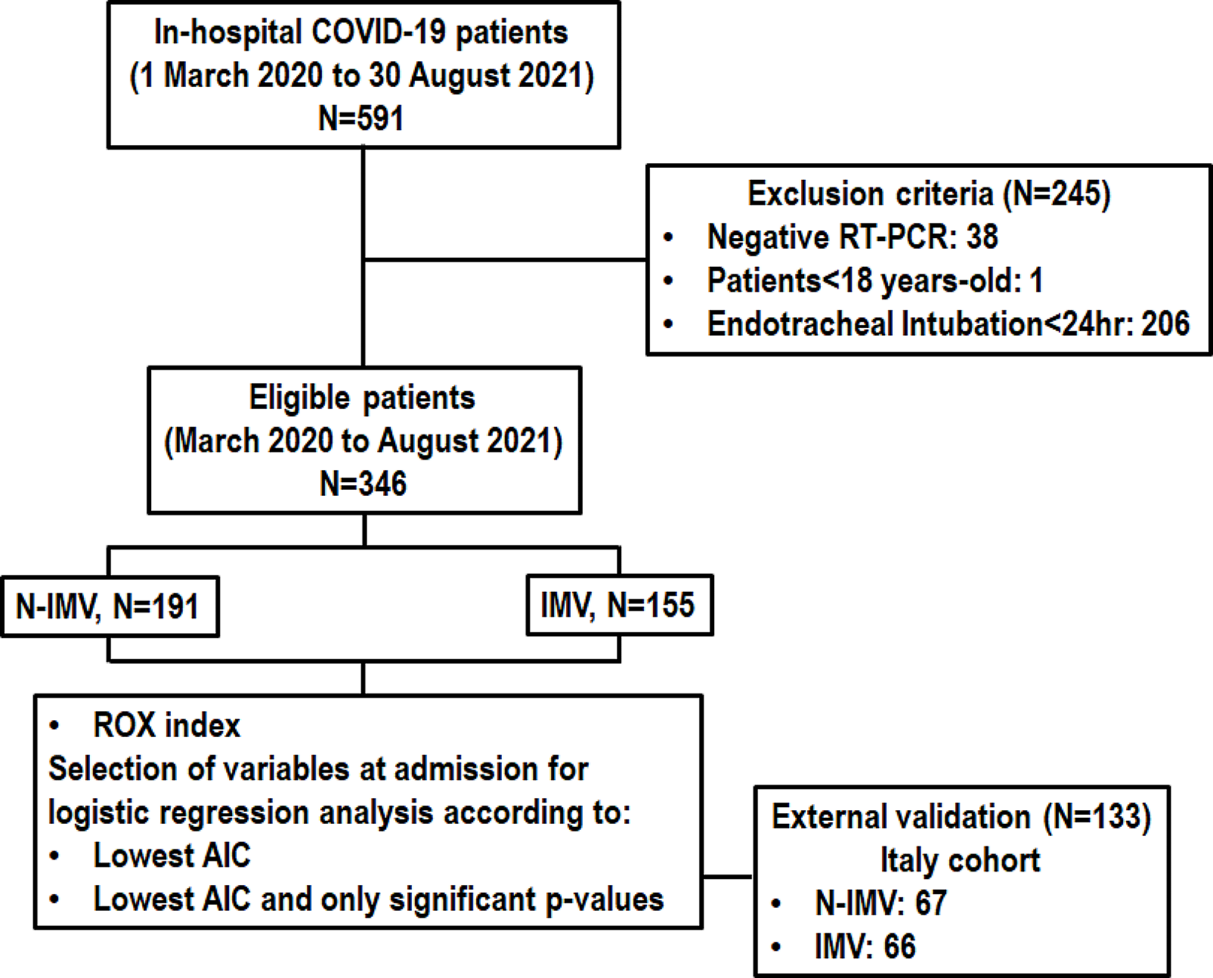
Flowchart of the study. AIC, Akaike Information Criteria; IMV, patients who were intubated and mechanically ventilated; N-IMV, patients who were not intubated and mechanically ventilated; ROX index, Respiratory rate-OXygenation index; RT-PCR, reverse transcriptase polymerase chain reaction

From March 2020 to August 2021, 591 ICU patients were screened and 346 were considered eligible (Fig. 1). Among them, 191 patients were not intubated and mechanically ventilated (N-IMV), and 155 received IMV. The median age of the ICU patients was 65 years (IQR, 53–73 years), with a median of 7 days (IQR, 5–10 days) of symptoms and 59.8% were male. Hypertension and diabetes mellitus were the most common comorbidities (55.5% and 33.0%, respectively). No significant differences were observed regarding age, days of symptoms, sex, and comorbidities between those in the IMV and N-IMV groups (Table 1).

**Table 1:** Characteristics of the development population at hospital admission

	No	All patients	*N*-IMV	IMV	*p* value between groups*
Absolute and relative frequencies, *n* (%)		346	191 (55.2)	155 (44.8)	
Age (years), median [IQR]	338	65 [53–73]	64 [53–73]	65 [54–73]	0.344
Duration of symptoms (days), median [IQR]	270	7 [5–10]	7 [5–12]	7 [5–10]	0.997
Sex, *n* (%)	346				
Male		207 (59.83)	117 (61.26)	90 (58.06)	0.624
Female		139 (40.17)	74 (38.74)	65 (41.94)	
Comorbidities, *n* (%)					
Hypertension	341	192 (55.49)	102 (54.84)	90 (58.06)	0.625
Diabetes mellitus	341	114 (32.95)	57 (30.65)	57 (36.77)	0.280
Obesity	338	39 (11.27)	21 (11.29)	18 (11.84)	1
Kidney failure	337	36 (10.4)	21 (11.29)	15 (9.93)	0.823
HIV	339	12 (3.47)	7 (3.76)	5 (3.27)	1
COPD	339	27 (7.8)	17 (9.14)	10 (6.54)	0.497
SOFA score, median [IQR]	317	2 [2–3]	2 [2–3]	3 [2–3]	< 0.001
SBP (mmHg), median [IQR]	323	128 [114–143]	130 [116–144]	121 [113–141]	0.030
DBP (mmHg), median [IQR]	325	77 [65–85]	75 [66–84]	78.5 [65–87]	0.235
HR (bpm), median [IQR]	325	87 [77–98]	87.5 [76–98]	87 [77–97]	0.773
Febrile, *n* (%)	323	24 (6.65)	16 (9.36)	8 (5.26)	0.235
SpO_2_ (%), median [IQR]	321	95 [91–97]	96 [94–98]	92 [90–96]	< 0.001
RR (bpm), median [IQR]	284	23 [20–26]	23 [18–26]	24 [22–27]	0.022
ROX, median [IQR]	177	8.5 [6.3–11.9]	9.6 [6.9–12.9]	7.3 [5.2–9.8]	< 0.001
Signs of breathing effort, *n* (%)	333	85 (24.57)	20 (11.14)	65 (41.94)	< 0.001
Hematocrit (%), median [IQR]	290	36.4 [30.6–40.0]	37.3 [32.6–41.5]	35.3 [29.4–39.2]	0.030
Leukocytes (cells/µL), median [IQR]	295	9400 [6475–12,750]	8100 [5815–10,993]	12,150 [7920–13,990]	< 0.001
Lymphocytes (cells/µL), median [IQR]	285	12 [8–16]	12 [8–18]	12 [7–14]	0.256
Platelets (10^6^/µL), median [IQR]	293	198 [150–280]	227 [168–316]	175 [145–247]	< 0.001
Na^+^ (mEq/L), median [IQR]	279	137 [134–140.5]	137 [133.25–140]	136 [134–141]	0.808
K^+^ (mEq/L), median [IQR]	280	4.1 [3.7–4.6]	4.3 [3.9–4.8]	4 [3.7–4.3]	< 0.001
Urea (mg/dL), median [IQR]	277	58 [35–86]	46 [32–74]	70 [44–103]	< 0.001
Creatinine (mg/dL), median [IQR]	289	1.10 [0.82–1.69]	1.10 [0.81–1.69]	1.18 [0.88–1.67]	< 0.001
C-reactive protein (mg/L), median [IQR]	267	48 [21–111]	48 [13–111]	48 [24–110]	0.374

The descriptive analysis of the data is presented as absolute frequencies (*n*) and percentages according to the group. No, the number of values gathered according to the respective variables; N-IMV, patients who were not endotracheally intubated and under invasive mechanical ventilation; IMV, patients who were endotracheally intubated and under invasive mechanical ventilation; IQR, interquartile range; HIV, human immunodeficiency virus; COPD, chronic obstructive pulmonary disease; SOFA, Sequential Organ Failure Assessment; SBP, systolic blood pressure; DBP, diastolic blood pressure; HR, heart rate; SpO_2_, peripheral oxygen saturation; RR, respiratory rate; ROX, Respiratory rate-OXygenation (ROX) index

*Mann-Whitney U test, Student’s t test or χ^2^ test (*p* < 0.05)

Several clinical and laboratory parameters differed significantly between the IMV and N-IMV groups. Notably, the IMV group had a higher SOFA score (median [interquartile range]: 3 [2, 3] versus 2 [2, 3], respectively; *p* < 0.001), lower systolic blood pressure (*p* = 0.030), lower SpO_2_ (*p* < 0.001), higher respiratory rate (*p* = 0.022), and increased signs of respiratory effort (41.9% versus 11.1%, *p* < 0.001). The ROX index at admission was lower in the IMV group than in the N-IMV group (7.29 [5.2–9.8] versus 9.64 [6.8–12.9], respectively; *p* = 0.001). In addition, leukocytes (*p* < 0.001), urea (*p* < 0.001), and creatinine (*p* < 0.001) levels were higher, whereas hematocrit (*p* = 0.030), platelets (*p* < 0.001), and potassium (*p* < 0.001) levels were lower in the IMV group. No differences in sodium and C-reactive protein were observed (Table 1). The hospital and ICU lengths of stay were higher in the IMV group (17 days [8–32 days] and 12 days [6–23 days]) than in the N-IMV group (12 days [8–21 days] and 7 days [4–11] days]; *p* = 0.031 and *p* < 0.001, respectively) (Supplementary Table 1). The mortality rate was higher in the IMV group than in the N-IMV group (73.5% versus 20.9%, *p* < 0.001).

### Characteristics of the COVID-19 external validation population

Of the 133 ICU patients in the external validation population, 67 were in the N-IMV group and 66 were in the IMV group (Supplementary Table 2). The median age of the patients was 61 years (range, 56–68 years), and 71.43% were male. Hypertension and obesity were the most common comorbidities (46.62% and 38.98%, respectively). There were more male patients than female patients in the IMV group (*p* = 0.015). However, no differences were observed in age or proportion of comorbidities between the N-IMV and IMV groups.

Several significant differences in clinical and laboratory parameters were noted between the IMV and N-IMV groups. The IMV group had a higher SOFA score (3 [2, 3] versus 2 [2, 3], respectively; *p* < 0.001) and a lower systolic blood pressure (*p* = 0.035) compared with the N-IMV group. In addition, the IMV group had a higher febrile status (*p* = 0.014), lower SpO_2_ (*p* = 0.001), and higher respiratory rate (*p* = 0.002). The proportion of signs of respiratory effort was also higher in the IMV group (80.3%) than in the N-IMV group (61.3%, *p* = 0.030). In terms of laboratory data, leukocytes were higher, whereas lymphocytes and platelets were lower in the IMV group compared with the N-IMV group (*p* < 0.001, *p* = 0.034, and *p* = 0.034, respectively). Sodium levels were lower, whereas creatinine and C-reactive protein levels were higher in the IMV group (*p* = 0.0004, *p* = 0.028, and *p* < 0.001, respectively). No differences were observed in hematocrit, potassium, or urea levels between the two groups.

### Multiple logistic regression analysis

The multiple logistic regression models identified several predictor variables associated with the need for IMV in ICU patients with COVID-19. The multiple logistic regression models with the lowest AIC (129.1) revealed the following predictor variables associated with IMV as the dependent variable (OR [95% CI]): arterial hypertension (2.61 [0.89–8.28]), diabetes mellitus (3.29 [1.11–10.73]), obesity (0.21 [0.04–0.99]), SOFA (1.69 [1.17–2.49]), heart rate (0.97 [0.94–1.00]), respiratory rate (1.07 [0.98–1.19]), SpO_2_ (0.83 [0.72–0.92]), febrile status (40.70 [1.08–1771.56]), signs of breathing effort (6.24 [1.82–24.51]), leukocytes (1.00 [1.00–1.00]) (Table 2 and Supplementary Table 3). In addition, the combination of the lowest AIC with significant *p* values revealed the following predictor variables associated with IMV as the dependent variable (OR [95% CI]): SOFA (1.46 [1.07–2.05]), SpO_2_ (0.81 [0.72–0.90]), signs of breathing effort (9.13 [3.29–28.67]) (Table 2 and Supplementary Table 3).

**Table 2:** Odds ratios and 95% confidence intervals of predictor variables recorded at hospital admission according to the lowest AIC and lowest AIC with only significant *p* values models to detect invasive mechanical ventilation

	Odds ratio [95% confidence interval]	*p* value
**Lowest AIC**		
Arterial hypertension	2.61 [0.89–8.28]	0.087
Diabetes mellitus	3.29 [1.11–10.73]	0.038
Obesity	0.21 [0.04–0.99]	0.062
SOFA score	1.69 [1.17–2.49]	0.006
HR	0.97 [0.94–1.00]	0.078
RR	1.07 [0.98–1.19]	0.148
SpO_2_	0.83 [0.72–0.92]	0.002
Febrile	40.70 [1.08–1771.56]	0.033
Signs of breathing effort	6.24 [1.82–24.51]	0.005
Leukocytes	1.00 [1.00–1.00]	0.004
**Lowest AIC combined with significant** ***p*** **values**		
SOFA score	1.46 [1.07–2.05]	0.020
SpO_2_	0.81 [0.72–0.90]	< 0.001
Signs of breathing effort	9.13 [3.29–28.67]	< 0.001

SOFA, Sequential Organ Failure Assessment; HR, heart rate; RR, respiratory rate; SpO_2_, peripheral oxygen saturation

### Assessment of the predictive performance for the ROX index and prognostic models

**Fig. 2:**
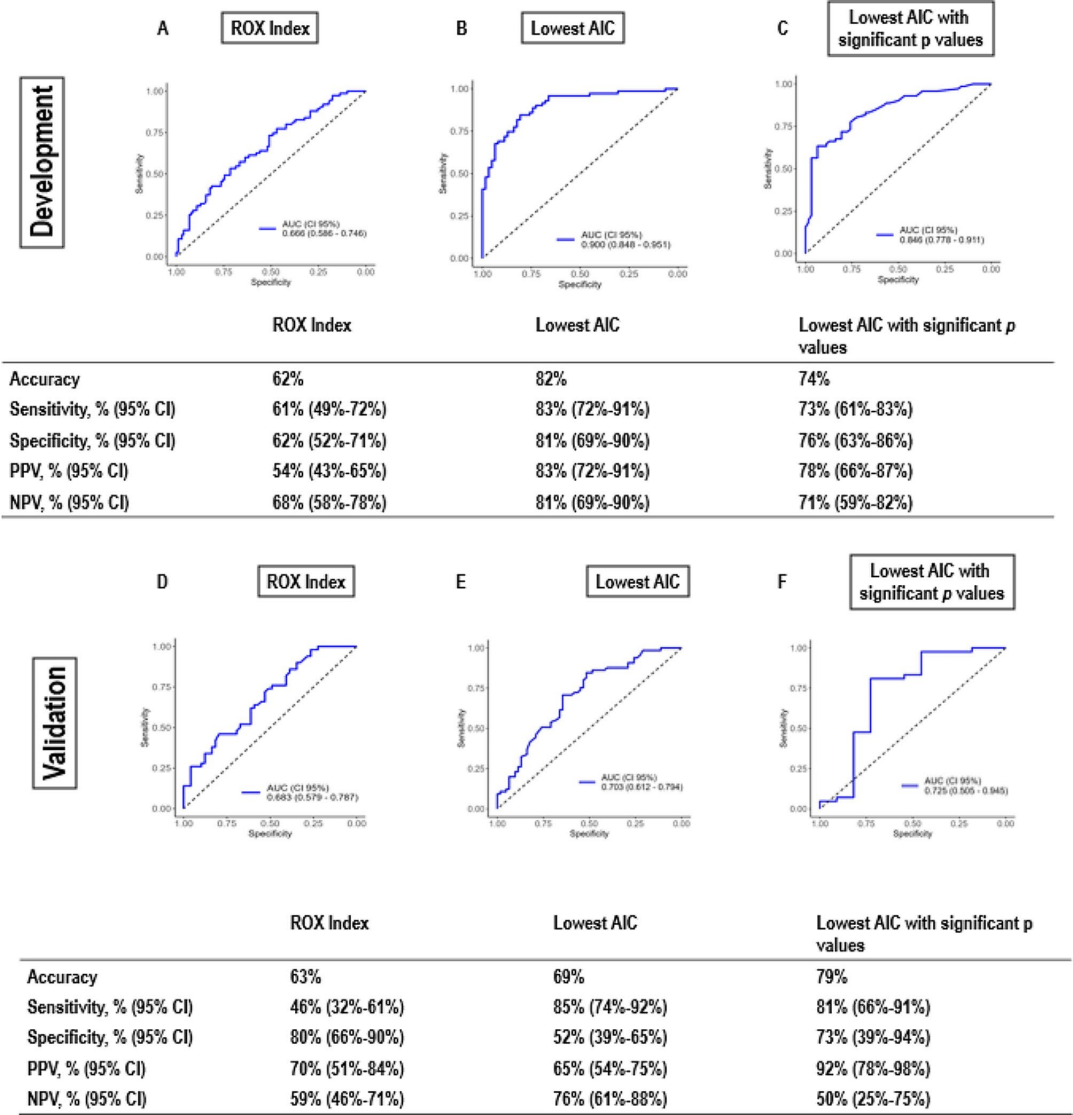
Assessment of the predictive performance for both prognostic models (AIC and AIC with significant *p* values) and the ROX index obtained from the development and external validation populations. (**A**–**C**) From the development population; (**C**–**E**) from external validation. AIC, Akaike Information Criteria; NPV, negative predictive value; PPV, positive predictive value; ROX, Respiratory rate-OXygentation index. AUCs were compared by DeLong’s algorithm. At the development population, the AUC of lowest AIC with significant p values model showed higher AUC compared with lowest AIC model and ROX index (*p* = 0.015, and *p* = 0.001, respectively). In addition, the lowest AIC model showed higher AUC than ROX index (*p* < 0.001). At the validation population, the AUC did not differ among ROX index, lowest AIC and lowest AIC with significant p values. The lowest AIC model was different between development and validation population (B vs. E, *p* < 0.001). However, the lowest AIC with significant p values model did not differ between development and validation population (C vs. F, *p* = 0.312)

The performance metrics for the ROX index and different models in the development population were as follows: ROX index: AUC, 0.666; accuracy, 62%; sensitivity, 61%; specificity, 62%; positive predictive value (PPV), 54%; negative predictive value (NPV), 68%. Lowest AIC model: AUC, 0.900; accuracy, 82%; sensitivity, 83%; specificity, 81%; PPV, 83%; NPV, 81%. Lowest AIC model with only significant *p* values: AUC, 0.846; accuracy, 74%; sensitivity, 73%; specificity, 76%; PPV, 78%; NPV, 71%. In the external validation population, the performance metrics for the ROX index and different models were as follows: ROX index: AUC, 0.683; accuracy, 63%; sensitivity, 46%; specificity, 80%; PPV, 70%; NPV, 59%. Lowest AIC model: AUC, 0.703; accuracy, 69%; sensitivity, 85%; specificity, 52%; PPV, 65%; NPV, 76%. Lowest AIC model with significant *p* values: AUC, 0.725; accuracy, 79%; sensitivity, 81%; specificity, 73%; PPV, 92%; NPV, 50% (Fig. 2). In the development population, the models based on the lowest AIC show better accuracy, sensitivity, and specificity compared with the ROX index, indicating their superiority in predicting IMV. In addition, the models with significant *p* values maintain good predictive performance while potentially simplifying the model by including fewer variables. In the validation population, the AUCs did not differ, but the lowest AIC and lowest AIC with significant p-values models maintained better values of accuracy and sensitivity compared to the ROX index. The lowest AIC model showed significant differences between the development and validation populations (*p* < 0.001). However, the lowest AIC with significant p-values model did not differ between the development and validation populations (*p* = 0.312).

## Discussion

The findings of the present study highlight several key points regarding predictors of the need for IMV in ICU patients with COVID-19. (1) Predictors identified by AIC: arterial hypertension, diabetes mellitus, obesity, SOFA score, heart rate, respiratory rate, SpO_2_, temperature, respiratory effort signals, and leukocytes were identified as predictors of IMV based on the AIC. (2) When AIC was combined with significant *p* values, SOFA score, SpO_2_, and respiratory effort signals emerged as the best predictors for IMV. This suggests that these variables have strong predictive value for the need for IMV in ICU patients with COVID-19. (3) Performance of the ROX index: the ROX index at admission was found to be lower in the IMV group than in the N-IMV group, indicating its potential usefulness as a predictor of IMV. However, its accuracy was lower compared with both prognostic models developed in the study. (4) Comparison of the models: in the development population, the receiver operating characteristic curve analysis demonstrated that the model based on the lowest AIC with significant *p* values outperformed both the ROX index and the model based on the lowest AIC alone in terms of AUC and accuracy. In the validation population, although the area under curve did not differ significantly, the lowest AIC and lowest AIC with significant p-values models demonstrated better accuracy and sensitivity compared to the ROX index. This suggests that SOFA score, SpO_2_, and respiratory effort signals may be robust predictors of the need for IMV in ICU patients with COVID-19. Overall, the study provides valuable insights into the predictors of IMV in ICU patients with COVID-19 and underscores the importance of incorporating multiple clinical and physiologic variables into predictive models for better accuracy and reliability.

The inclusion and exclusion criteria of the study were carefully designed to ensure a specific and relevant patient population for the investigation of predictive models for IMV in COVID-19 patients. Patients aged 18 years and older admitted to the ICU with a confirmed diagnosis of COVID-19 were included in the study. This broad inclusion criterion was aimed at capturing a comprehensive range of adult patients experiencing severe COVID-19 symptoms, thereby ensuring the study results would be widely applicable to the adult ICU population affected by the pandemic [18, 19]. Patients who were mechanically ventilated within the first 24 h of ICU admission were excluded from the study. The rationale behind this exclusion criterion is: (1) Data collection period: The first 24 h were deemed the minimum period necessary to gather sufficient data to understand the clinical evolution of patients. This timeframe allows for the collection of essential clinical parameters and the assessment of the patient’s condition beyond the immediate emergency response; (2) Clinical decision-making: Immediate intubation within the first 24 h often indicates a rapidly deteriorating condition, necessitating urgent intervention. Including these patients could skew the data, as their clinical trajectory and immediate need for intubation might differ significantly from those whose condition evolves more gradually; and (3) Homogeneity of the study population: Excluding patients intubated within the first 24 h helps to create a more homogeneous study population, focusing on those whose need for intubation arises after an initial period of ICU management. This can provide clearer insights into the predictive factors and clinical markers that emerge within the first 24 h and influence the subsequent need for IMV [20, 21]. While the criteria are justified, it is also important to recognize potential limitations. The exclusion of early intubation cases may omit a subset of patients with severe disease progression, potentially leading to an underestimation of certain risk factors. Moreover, as the emergency department and the ICU are not part of the same institution, there could be variability in the initial assessment and management practices, which might affect the generalizability of the findings. Overall, these criteria are aligned with the study’s objective to create a robust predictive model for IMV in COVID-19 patients, focusing on those whose clinical deterioration necessitates intubation beyond the immediate critical period. Despite having significant resources, including ventilators and critical care beds, several countries still face challenges in ensuring adequate availability of mechanical ventilators for all patients in need [22]. Existing scoring systems for predicting respiratory failure and the need for mechanical ventilation have limitations, including small sample sizes and low predictive power. Frontline healthcare providers have emphasized the urgent need for the development of new warning systems to identify patients for whom non-invasive respiratory support is likely to fail and who will require mechanical ventilation [4, 6]. During the first year of the COVID-19 pandemic, most prediction models were unclear or had a high risk of bias [21]. Although external validations of these models were conducted, some still lacked independent validation and showed a high risk of bias. The proliferation of insufficiently validated models may not be useful for clinical practice and could potentially cause harm if relied upon inaccurately [7, 8, 24]. The ROX index, primarily used to predict failure of HFNO in patients with acute respiratory failure [25], has shown moderate predictive ability in various studies [26, 27]. However, its accuracy in predicting failure of NIV is somewhat limited. Combining the ROX index with other relevant variables may enhance its predictive performance for the need for IMV.

The SOFA score has been widely used in critical care settings to assess organ dysfunction and predict outcomes in critically ill patients. In the context of COVID-19 pneumonia and acute respiratory distress syndrome, the SOFA score has been shown to be useful in predicting mortality and guiding clinical management [28]. Fayed et al. [29] demonstrated that the SOFA score had a high discriminatory ability (AUC, 0.883) for predicting mortality in patients with acute respiratory distress syndrome associated with COVID-19. This indicates that the SOFA score is effective in identifying patients at higher risk of mortality, allowing healthcare providers to intervene appropriately and allocate resources effectively.

Changes in oxygenation indices (OIs) and risk scores have been evaluated in a retrospective study of diagnostic tests of 1,402 patients hospitalized with COVID-19. PaO_2_/FiO_2_, 4 C mortality score, SOFA score, and SaO_2_/FiO_2_ were weak predictors of the need for mechanical ventilation from admission [30]. This is attributed to the fact that receiver operating characteristic curves were independently calculated for each of the OIs and risk indices [31]. Therefore, conducting integrated assessments that consider both OIs and risk indices may be essential to estimate damage across various organs or systems, as commonly observed in severe pneumonia due to COVID-19 [32]. Cattazzo et al. [33] showed that the ROX index, compared with the PaO_2_/FiO_2_ ratio and the SaO_2_/FiO_2_ ratio, better predicted the need for mechanical ventilation in 456 patients hospitalized due to COVID-19. In our study, the ROX index was lower in the ICU patients on IMV in both the development and validation populations. However, using components of the ROX index (SpO_2_, signs of breathing effort) and associating it with the SOFA score, a better performance in predicting the need for mechanical ventilation was observed in the validation population. The lowest AIC model reached the best accuracy for the development population. However, this model needs several pieces of information, such as the presence of arterial hypertension, diabetes mellitus, and obesity, the SOFA score, heart rate, respiratory rate, SpO_2_, febrile status, signs of breathing effort, leukocytes, which can be time-consuming and not practical at the bedside. In addition, the simple prognostic model showed a better accuracy and sensitivity in the validation population and could be easily applied at the bedside even in facilities with limited resources. Garcia-Gordillo et al. [34] found that the biomarkers used in the COVID-Intubation Risk Score (respiratory rate, SaO_2_/FiO_2_ ratio, lactate dehydrogenase level, and either interleukin-6 or neutrophil/lymphocyte ratio), accurately represent relevant aspects of the clinical phenomena seen in severe COVID-19. Both the respiratory rate and the SaO_2_/FiO_2_ ratio evaluate ventilatory function and its deterioration is the main component associated with IMV in patients with COVID-19 [35].

The present study has some limitations. First, missing values were closely monitored. During the early phase of the pandemic, some information was missing. Nevertheless, in the multiple regression models, we only used variables when the proportion of missing values was < 25% of the total. Second, we would like to test classic predictive scores for mechanical ventilation, such as the HACOR (heart rate, acidosis, consciousness, oxygenation, respiratory rate) score. However, we did not have data on the Glasgow Coma Scale, which would jeopardize the HACOR values. Third, the sample size calculation was not performed specifically for stratified analyses. To conduct a stratified analysis for variables like obesity and diabetes mellitus, more patients would be necessary. However, both obesity and diabetes mellitus were collected and included in the lowest AIC model. Forth, we cannot exclude the possibility that important variables, such as radiological findings, biological markers, time under non-invasive ventilation (NIV) or high-flow nasal oxygen (HFNO), diaphragm ultrasound measures, and surrogate markers of muscle activity [(airway occlusion pressure (P_0.1_) and expiratory occlusion pressure (Pocc)], which could influence regression analysis, were not initially considered. Nevertheless, we opted to use a simple and practical predictive score for mechanical ventilation, such as the ROX index. The ROX index has been validated in HFNO devices [25], but it has also been used in cases of NIV [26].

## Conclusions

In ICU patients with COVID-19, the SOFA score, SpO_2_, and respiratory effort signals demonstrated superior performance in predicting the need for IMV compared to the ROX index in the development population. In the external validation population, while the AUCs did not show significant differences, the accuracy was notably higher using SOFA score, SpO_2_, and respiratory effort signals when compared with the ROX index. This suggests that these variables are more effective in predicting the need for IMV in ICU patients with COVID-19.

### Electronic supplementary material

Below is the link to the electronic supplementary material.

Supplementary Material 1

Supplementary Material 2

Supplementary Material 3

## Data Availability

The datasets used and/or analyzed during the current study are available from the corresponding author on reasonable request.

## Abbreviations

AIC: Akaike Information Criteria
AUC: area under the curve
CI: confidence interval
HACOR: heart rate, acidosis, consciousness, oxygenation, respiratory rate
HFNO: high-flow nasal oxygen
ICU: intensive care unit
IMV: invasive mechanical ventilation
IQR: interquartile range
NIV: non-invasive ventilation
N-IMV: not intubated and mechanically ventilated
NPV: negative predictive value
OI: oxygenation index
OR: odds ratio
P_01_: airway occlusion pressure
Pocc: expiratory occlusion pressure
PPV: positive predictive value
ROX: Respiratory rate-OXygenation
SOFA: Sequential Organ Failure Assessment
SpO_2_: peripheral oxygen saturation
