# Supplementary material for: Derivation and external validation of predictive models for invasive mechanical ventilation in intensive care unit patients with COVID-19
Source: Ann Intensive Care. 2024 Aug 21;14:129. doi: 10.1186/s13613-024-01357-4 (PMC11339005; doi:10.1186/s13613-024-01357-4)
Supplement: Supplementary file 1 — Supplementary Material 1 [file 13613_2024_1357_MOESM1_ESM.docx]

**Supplementary Table 1** Primary and secondary outcomes of development population

|  | **All patients (*n*=346)** | **N-IMV (*n*=191)** | **IMV (*n*=155)** | ***P* value between groups*** |
| --- | --- | --- | --- | --- |
| IMV rate, % (CI95%) | 44.8 (39.5 – 50.2) | - | - | - |
| Hospital length of stay (days), median [IQR] | 13 [8–26] | 12 [8–21] | 17 [8–32] | 0.031 |
| ICU length of stay (days), median [IQR] | 8 [5–17] | 7 [4–11] | 12 [6–23] | <0.001 |
| Time to IMV, (days), median [IQR] | - | - | 2 [1–3] | - |
| Time under IMV (days), median [IQR] | - | - | 9 [4–18] | - |
| Mortality rate, % (CI95%) | 44.5 (39.2 – 49.9) | 20.9 (15.5 – 27.5) | 73.5 (65.8 – 80.2) | <0.001 |

N-IMV, not under invasive mechanical ventilation; IMV, invasive mechanical ventilation; IQR, interquartile range; ICU, intensive care unit. CI95%: Confidence Interval 95%

*Mann-Whitney U test, Student’s t test or χ^2^ test (*p*<0.05).
