# Supplementary material for: Derivation and external validation of predictive models for invasive mechanical ventilation in intensive care unit patients with COVID-19
Source: Ann Intensive Care. 2024 Aug 21;14:129. doi: 10.1186/s13613-024-01357-4 (PMC11339005; doi:10.1186/s13613-024-01357-4)
Supplement: Supplementary file 2 — Supplementary Material 2 [file 13613_2024_1357_MOESM2_ESM.docx]

**Supplemental Table 2** Characteristics of the external validation population at hospital admission (Italian database)

|  | **No** | **All patients** | **N-IMV** | **IMV** | ***p* value between groups*** |
| --- | --- | --- | --- | --- | --- |
| Absolute and relative frequencies, *n* (%) |  | 133 | 67 (50.4) | 66 (49.6) |  |
| Age (years), median [IQR] | 133 | 61 [56–68] | 62 [56–70] | 60 [56–67] | 0.053 |
| Sex, *n* (%) | 133 |  |  |  |  |
| Male |  | 95 (71.43) | 41 (61.19) | 54 (81.82) | 0.015 |
| Female |  | 38 (28.57) | 26 (38.81) | 12 (18.18) |  |
| Comorbidities, *n* (%) |  |  |  |  |  |
| Hypertension | 133 | 62 (46.62) | 33 (49.25) | 29 (43.94) | 0.660 |
| Diabetes mellitus | 133 | 20 (15.04) | 11 (16.42) | 9 (13.64 ) | 0.837 |
| Obesity | 59 | 23 (38.98) | 4 (30.77) | 19 (41.30) | 0.715 |
| Kidney failure | 133 | 1 (0.75) | 1 (1.49) | 0 (0) | 1.000 |
| HIV | 1 | 1 (100) | 1 (100) | 0 (0) | - |
| COPD | 133 | 5 (3.76) | 2 (2.99) | 3 (4.55) | 0.986 |
| SOFA score, median [IQR] | 133 | 3 [2–3] | 2 [2–3] | 3 [2–3] | <0.001 |
| SBP (mmHg), median [IQR] | 132 | 140 [125–153] | 140 [131–159] | 138 [120–150] | 0.035 |
| DBP (mmHg), median [IQR] | 132 | 80 [70–90] | 80 [74–90] | 80 [70–90] | 0.249 |
| HR bpm), median [IQR] | 128 | 95 [85–106] | 90 [81–104] | 98 [88–106] | 0.053 |
| Febrile, *n* (%) | 132 | 72 (54.55) | 29 (43.28) | 43 (66.15) | 0.014 |
| SpO_2_ (%), median [IQR] | 132 | 91 [87–95] | 93 [89–97] | 89 [80–93] | 0.001 |
| RR (bpm), median [IQR] | 114 | 20 [16–24] | 19 [15–22] | 21 [18–27] | 0.002 |
| ROX index, median [IQR] | 113 | 21.2 [15.8–26.5] | 23.3 [19.3–29.8] | 20.2 [14.1–23.5] | <0.001 |
| Signs of breathing effort, *n* (%) | 128 | 91 (71.09) | 38 (61.29) | 53 (80.30) | 0.030 |
| Hematocrit (%), median [IQR] | 133 | 41.2 [38.5–43.5] | 40.5 [37–44.2] | 35.3 [29.4–39.2] | 0.271 |
| Leukocytes (cells/μL), median [IQR] | 133 | 7,380 [5,560–10,320] | 7,210 [5,020–9,640] | 7,390 [5,740–11,330] | <0.001 |
| Lymphocytes (cells/μL), median [IQR] | 133 | 13.4 [8.1–20] | 14.4 [8.85–22.55] | 11.8 [7.43–17.77] | 0.034 |
| Platelets (10^6^/μL), median [IQR] | 133 | 191 [158–262] | 206 [159–262] | 187 [155–261] | 0.034 |
| Na^+^ (mEq/L), median [IQR] | 132 | 137 [135–140] | 138 [136–141] | 136 [134–139] | 0.004 |
| K^+^ (mEq/L), median [IQR] | 132 | 4.05 [3.8–4.4] | 4.1 [3.8–4.4] | 4 [3.8–4.3] | 0.579 |
| Urea (mg/dL), median [IQR] | 133 | 35 [27–50] | 33 [24–46] | 38 [27–52] | 0.288 |
| Creatinine (mg/dL), median [IQR] | 133 | 1 [0.80–1.20] | 1 [0.80–1.10] | 1.10 [0.90–1.30] | 0.028 |
| C-reactive protein, median [IQR], mg/L | 132 | 8.02 [3.92–15.64] | 6.15 [3.01–9.68] | 11.39 [6.81–19.95] | <0.001 |

The descriptive analysis of the data is presented as absolute frequencies (*n*) and percentages according to the group. No, the number of values gathered according to the respective variables; N-IMV, patients who were not endotracheally intubated and under invasive mechanical ventilation; IMV, patients who were endotracheally intubated and under invasive mechanical ventilation; IQR, interquartile range; HIV, human immunodeficiency virus; COPD, chronic obstructive pulmonary disease; SOFA, Sequential Organ Failure Assessment; SBP, systolic blood pressure; DBP, diastolic blood pressure; HR, heart rate; SpO_2_, peripheral oxygen saturation; RR, respiratory rate; ROX, Respiratory rate-OXygenation (ROX) index.

*Mann-Whitney U test, Student’s t test or χ^2^ test (*p*<0.05).
