# Supplementary material for: Derivation and external validation of predictive models for invasive mechanical ventilation in intensive care unit patients with COVID-19
Source: Ann Intensive Care. 2024 Aug 21;14:129. doi: 10.1186/s13613-024-01357-4 (PMC11339005; doi:10.1186/s13613-024-01357-4)
Supplement: Supplementary file 3 — Supplementary Material 3 [file 13613_2024_1357_MOESM3_ESM.docx]

**Supplementary Table 3** Multiple logistic regression models by the traditional method of input, initially with all variables and removing the least significant (or the one with the lowest coefficient), according to IMV as the dependent variable (yes or no)

| **Models** | **1** | **2** | **3** | **4** | **5** | **6** | **7** | **8** | **9** | **10** | **11** | **12** | **13** | **14** | **15** | **16** | **17** | **18** | **19** | **20** | **21** | **22** | **23** | **24** |
| --- | --- | --- | --- | --- | --- | --- | --- | --- | --- | --- | --- | --- | --- | --- | --- | --- | --- | --- | --- | --- | --- | --- | --- | --- |
| (Intercept) | 0.03 | 0.02 | 0.02 | 0.02 | 0.02 | 0.02 | 0.02 | 0.02 | 0.02 | 0.02 | 0.02 | 0.03 | 0.03 | 0.05 | 0.05 | 0.03 | 0.03 | 0.01 | 0.03 | 0.02 | 0.03 | 0.03 | 0.02 | 0.00 |
| Age | 0.76 | 0.75 | 0.75 | 0.76 | 0.74 | 0.74 | 0.76 |  |  |  |  |  |  |  |  |  |  |  |  |  |  |  |  |  |
| Days of symptoms | 0.85 | 0.86 | 0.85 | 0.84 | 0.85 |  |  |  |  |  |  |  |  |  |  |  |  |  |  |  |  |  |  |  |
| Male sex | 0.96 | 0.95 |  |  |  |  |  |  |  |  |  |  |  |  |  |  |  |  |  |  |  |  |  |  |
| Arterial hypertension | 0.11 | 0.10 | 0.10 | 0.10 | 0.10 | 0.09 | 0.09 | 0.09 | 0.07 | 0.08 | 0.08 | 0.08 | 0.06 | 0.06 | 0.05 | 0.06 | 0.09 | 0.12 | 0.17 |  |  |  |  |  |
| Diabetes mellitus | 0.02 | 0.02 | 0.02 | 0.02 | 0.02 | 0.02 | 0.02 | 0.02 | 0.01 | 0.01 | 0.01 | 0.01 | 0.02 | 0.02 | 0.02 | 0.03 | 0.04 | 0.06 | 0.07 | 0.04 | 0.06 |  |  |  |
| Obesity | 0.04 | 0.04 | 0.04 | 0.04 | 0.04 | 0.04 | 0.04 | 0.04 | 0.05 | 0.04 | 0.05 | 0.05 | 0.08 | 0.09 | 0.06 | 0.06 | 0.06 | 0.07 | 0.06 | 0.06 | 0.06 | 0.06 |  |  |
| CKD | 0.16 | 0.16 | 0.16 | 0.13 | 0.13 | 0.13 | 0.12 | 0.13 | 0.08 | 0.11 | 0.10 | 0.09 | 0.14 | 0.13 | 0.11 | 0.17 |  |  |  |  |  |  |  |  |
| HIV | 0.40 | 0.40 | 0.39 | 0.39 | 0.40 | 0.41 | 0.40 | 0.38 | 0.36 | 0.37 |  |  |  |  |  |  |  |  |  |  |  |  |  |  |
| COPD | 0.85 | 0.85 | 0.85 | 0.85 | 0.85 | 0.83 |  |  |  |  |  |  |  |  |  |  |  |  |  |  |  |  |  |  |
| SOFA score | 0.01 | 0.01 | 0.00 | 0.00 | 0.00 | 0.00 | 0.00 | 0.00 | 0.00 | 0.00 | 0.00 | 0.00 | 0.00 | 0.01 | 0.01 | 0.00 | 0.01 | 0.01 | 0.02 | 0.01 | 0.02 | 0.03 | 0.05 | 0.02 |
| SBP | 0.37 | 0.36 | 0.35 | 0.35 | 0.35 | 0.36 | 0.36 | 0.33 | 0.31 | 0.26 | 0.29 |  |  |  |  |  |  |  |  |  |  |  |  |  |
| DBP | 0.15 | 0.15 | 0.15 | 0.15 | 0.15 | 0.15 | 0.15 | 0.14 | 0.13 | 0.13 | 0.15 | 0.31 |  | 0.10 |  |  |  |  |  |  |  |  |  |  |
| HR | 0.05 | 0.05 | 0.05 | 0.05 | 0.04 | 0.04 | 0.04 | 0.04 | 0.05 | 0.05 | 0.05 | 0.06 | 0.09 | 0.07 | 0.13 | 0.07 | 0.08 | 0.14 |  |  |  |  |  |  |
| RR | 0.08 | 0.07 | 0.07 | 0.07 | 0.07 | 0.06 | 0.06 | 0.06 | 0.06 | 0.05 | 0.06 | 0.08 | 0.10 | 0.00 | 0.08 | 0.13 | 0.15 |  |  |  |  |  |  |  |
| SpO_2_ | 0.00 | 0.00 | 0.00 | 0.00 | 0.00 | 0.00 | 0.00 | 0.00 | 0.00 | 0.00 | 0.00 | 0.00 | 0.00 | 0.03 | 0.00 | 0.00 | 0.00 | 0.00 | 0.00 | 0.00 | 0.01 | 0.01 | 0.01 | 0.00 |
| Febrile status | 0.08 | 0.08 | 0.08 | 0.08 | 0.07 | 0.06 | 0.04 | 0.05 | 0.05 | 0.06 | 0.05 | 0.03 | 0.04 | 0.02 | 0.04 | 0.03 | 0.03 | 0.06 | 0.06 | 0.10 |  |  |  |  |
| Signs of effort | 0.17 | 0.17 | 0.15 | 0.15 | 0.15 | 0.12 | 0.12 | 0.13 | 0.12 | 0.14 | 0.12 | 0.04 | 0.02 | 0.02 | 0.02 | 0.01 | 0.01 | 0.00 | 0.00 | 0.00 | 0.00 | 0.00 | 0.00 | 0.00 |
| Hematocrit | 0.97 |  |  |  |  |  |  |  |  |  |  |  |  |  |  |  |  |  |  |  |  |  |  |  |
| Leukocytes | 0.01 | 0.01 | 0.01 | 0.01 | 0.01 | 0.01 | 0.01 | 0.00 | 0.00 | 0.00 | 0.00 | 0.00 | 0.00 | 0.00 | 0.00 | 0.01 | 0.00 | 0.01 | 0.02 | 0.02 | 0.03 | 0.04 | 0.06 |  |
| Lymphocytes | 0.89 | 0.89 | 0.89 | 0.90 |  |  |  |  |  |  |  |  |  |  |  |  |  |  |  |  |  |  |  |  |
| Platelets | 0.28 | 0.28 | 0.28 | 0.28 | 0.25 | 0.24 | 0.23 | 0.24 | 0.15 | 0.15 | 0.15 | 0.12 | 0.13 | 0.20 | 0.16 |  |  |  |  |  |  |  |  |  |
| Na^+^ | 0.43 | 0.43 | 0.43 | 0.42 | 0.42 | 0.41 | 0.39 | 0.38 | 0.40 |  |  |  |  |  |  |  |  |  |  |  |  |  |  |  |
| K^+^ | 0.50 | 0.50 | 0.50 | 0.50 | 0.48 | 0.48 | 0.48 | 0.51 |  |  |  |  |  |  |  |  |  |  |  |  |  |  |  |  |
| Urea | 0.51 | 0.51 | 0.50 | 0.43 | 0.42 | 0.41 | 0.41 | 0.39 | 0.28 | 0.28 | 0.29 | 0.29 | 0.31 |  |  |  |  |  |  |  |  |  |  |  |
| Creatinine | 0.92 | 0.93 | 0.93 |  |  |  |  |  |  |  |  |  |  |  |  |  |  |  |  |  |  |  |  |  |
| C-reactive protein | 0.14 | 0.14 | 0.14 | 0.14 | 0.13 | 0.13 | 0.12 | 0.11 | 0.13 | 0.12 | 0.13 | 0.19 | 0.24 | 0.30 |  |  |  |  |  |  |  |  |  |  |
| AIC | 150.5 | 148.5 | 146.5 | 144.5 | 142.5 | 140.5 | 138.6 | 136.7 | 135.1 | 133.8 | 132.7 | 131.8 | 131.0 | 130.1 | 129.2 | 129.2 | 129.1 | 129.2 | 129.4 | 129.3 | 129.7 | 131.6 | 133.5 | 135.2 |

Model 17 achieved the lowest Akaike Information Criteria [AIC (129.1)] and Model 24 achieved the lowest Akaike Information Criteria with significant *p* values (most simple model). CKD, chronic kidney disease; HIV, human immunodeficiency virus; COPD, chronic obstructive pulmonary disease; SOFA, Sequential Organ Failure Assessment; SBP, systolic blood pressure; DBP, diastolic blood pressure; HR, heart rate; RR, respiratory rate; SpO_2_, peripheral oxygen saturation.

Model 17: Selected predictor variables: arterial hypertension, diabetes melliuts, obesity, SOFA, HR, RR, SpO_2_, febrile, signs of breathing effort, leukocytes.

Model 24: Selected predictor variables: SOFA, SpO_2_, signs of breathing effort.
